# Morphological Diversity between Culture Strains of a Chlorarachniophyte, *Lotharella globosa*

**DOI:** 10.1371/journal.pone.0023193

**Published:** 2011-08-15

**Authors:** Yoshihisa Hirakawa, Alexis Howe, Erick R. James, Patrick J. Keeling

**Affiliations:** Canadian Institute for Advanced Research and Department of Botany, University of British Columbia, Vancouver, Canada; University of British Columbia, Canada

## Abstract

Chlorarachniophytes are marine unicellular algae that possess secondary plastids of green algal origin. Although chlorarachniophytes are a small group (the phylum of Chlorarachniophyta contains 14 species in 8 genera), they have variable and complex life cycles that include amoeboid, coccoid, and/or flagellate cells. The majority of chlorarachniophytes possess two or more cell types in their life cycles, and which cell types are found is one of the principle morphological criteria used for species descriptions. Here we describe an unidentified chlorarachniophyte that was isolated from an artificial coral reef that calls this criterion into question. The life cycle of the new strain includes all three major cell types, but DNA barcoding based on the established nucleomorph ITS sequences showed it to share 100% sequence identity with *Lotharella globosa*. The type strain of *L. globosa* was also isolated from a coral reef, but is defined as completely lacking an amoeboid stage throughout its life cycle. We conclude that *L. globosa* possesses morphological diversity between culture strains, and that the new strain is a variety of *L. globosa*, which we describe as *Lotharella globosa* var. *fortis* var. nov. to include the amoeboid stage in the formal description of *L. globosa*. This intraspecies variation suggest that gross morphological stages maybe lost rather rapidly, and specifically that the type strain of *L. globosa* has lost the ability to form the amoeboid stage, perhaps recently. This in turn suggests that even major morphological characters used for taxonomy of this group may be variable in natural populations, and therefore misleading.

## Introduction

Chlorarachniophytes are marine unicellular photosynthetic organisms that have acquired their plastid via a secondary endosymbiosis between a green alga and a cercozoan protist [1], [2]. The plastid is surrounded by four envelope membranes and contains a highly reduced nucleus, referred to as the nucleomorph, of the green algal endosymbiont in the periplastidal compartment between the inner and outer pairs of envelope membranes [3], [4]. Chlorarachniophytes are widely distributed in marine environments from tropical coast to open ocean, and they possess various cell types such as amoeboid, coccoid, and flagellate cells. Most species possess two or more cell types and have specific transformations between types throughout their life cycles, but the number of types is variable from genus to genus. For example, *Lotharella vacuolata* and *Chlorarachnion reptans* possess all three cell types [5], [6], whereas *Bigelowiella natans* is only found in the flagellate form [7]. This variation is one of the cornerstones of chlorarachniophyte classification, the others being plastid ultrastructure (pyrenoid structure and the position of the nucleomorph in the periplastidal compartment) and morphological characters of the main vegetative cells [1]. Recently, a DNA barcoding system has also been developed for rapid and accurate identification of chlorarachniophyte species based on the internal transcribed spacer (ITS) sequences of nuclear and nucleomorph rRNA cistrons [8].

Since the phylum Chlorarachniophyta was established by Hibberd and Norris in 1984 [6], only 14 species and 8 genera have been formally described. The large number of mono-specific genera is striking, and likely reflects the fact that many or all of the morphological characters used in taxonomy only distinguish genus-level differences, so many cryptic species could go unsampled. The genus *Lotharella* Ishida et Y. Hara is the biggest exception to this trend, since this genus contains five species, *L. globosa*, *L. polymorpha*, *L. vacuolata*, *L. oceanica*, and *L. reticulosa* [5], [9]–[12], [13]; recently *L. amoebiformis* was placed to a new genus, *Amorphochlora* Ishida, Yabuki et Ota [14]. Life cycle characters were mainly used for the classification of *Lotharella* species. For example, *L. globosa* and *L. oceanica* completely lack the amoeboid stage in their life cycles whereas *L. reticulosa*, *L. polymorpha*, and *L. vacuolata* possess the amoeboid stage, and the main vegetative stage of *L. oceanic* is naked spherical cells whereas the others are coccoid cells with single- or multi-layer cell walls [5], [12], [13]. Nucleomorph ITS barcode sequences support the distinction between all the *Lotharella* species, with the exception of *L. polymorpha*, for which no sequence data are available [8], [13].

In this study, we present morphological, ultrastructural, and molecular characterization of a new chlorarachniophyte strain, LEX01, which reveals the first major discrepancy between molecular and morphological species distinctions in this phylum. The result of DNA barcoding strongly suggested that LEX01 is a strain of *L. globosa*, since their nucleomorph ITS sequences are 100% identical (as are the sequences of nuclear small subunit rRNA). In contrast, however, LEX01 and the type strain of *L. globosa* do not share the same life history stages; whereas *L. globosa* completely lacks amoeboid stage in its life cycle, and this is one of the defining characteristics of this species, we observed numerous amoeboid cells in fresh cultures of the LEX01 strain. Based on traditional morphological characters, LEX01 would never be identified as *L. globosa*, but we conclude that it is, and that the type strain of *L. globosa* has lost a major life history stage, perhaps very recently. This has interesting implications for the stability of these characters in natural populations.

## Results

### Identification of LEX01 strain by DNA barcoding

Samples of coral mucus were isolated from an artificial coral reef at the Birch Aquarium (Scripps Institution of Oceanography, San Diego) and protists from the mucus were serially diluted to establish low-diversity cultures. From one dilution a chlorarachniophyte was identified, and a mono-eukaryote culture, named LEX01, established by manually picking a single cell. DNA was extracted from LEX01, and the SSU rRNA (accession number JF826444) and internal transcribed spacer (ITS) sequences (JF806440 to JF806448) from both the nuclear and nucleomorph genome were amplified by PCR using specific primer sets. The nucleomorph ITS is currently the only well sampled barcode marker for species-level distinctions available [8], so this locus from LEX01 was compared with barcode sequences from other chlorarachniophyte species. The full sequence (596 bases) of LEX01 was found to be 100% identical to the sequence of three *Lotharella globosa* strains (CCMP1729 is the type strain, and two other synonyms are CCMP2314 and CCCM811). LEX01 shared only 83.4% (473/567 base) identity with *L. reticulosa*, 82.7% (493/596 bases) with *L. oceanica* (CCMP622), and 75.2% (448/596 bases) with *L. vacuolata* (CCMP240), the other three species of *Lotharella* for which molecular data exist. Nuclear ITS sequences were obtained by cloning, since they have been shown to be variable within a monoculture [8]. Consistent with this, we detected three and five distinct sequences from LEX01 and CCCM811, respectively, but pairwise comparisons nevertheless showed LEX01 (JF806441 to JF806443) and *L. globosa* CCCM811 (JF806444 to JF806448) shared the greatest level of sequence identity, with 98.1 to 98.7% (1248 to 1256 per 1272 bases) between these two strains. The inter-locus variability of these ITS sequences was calculated to be 0.3–2.3% (4–29/1272 bases) and 0.4–1.0% (5–13/1272 bases) in LEX01 and CCCM811 strain, respectively. This indicates that the sequence difference between these two strains is within the range of the inter-locus variability.

### Life cycle and morphology of LEX01

**Figure 1:**
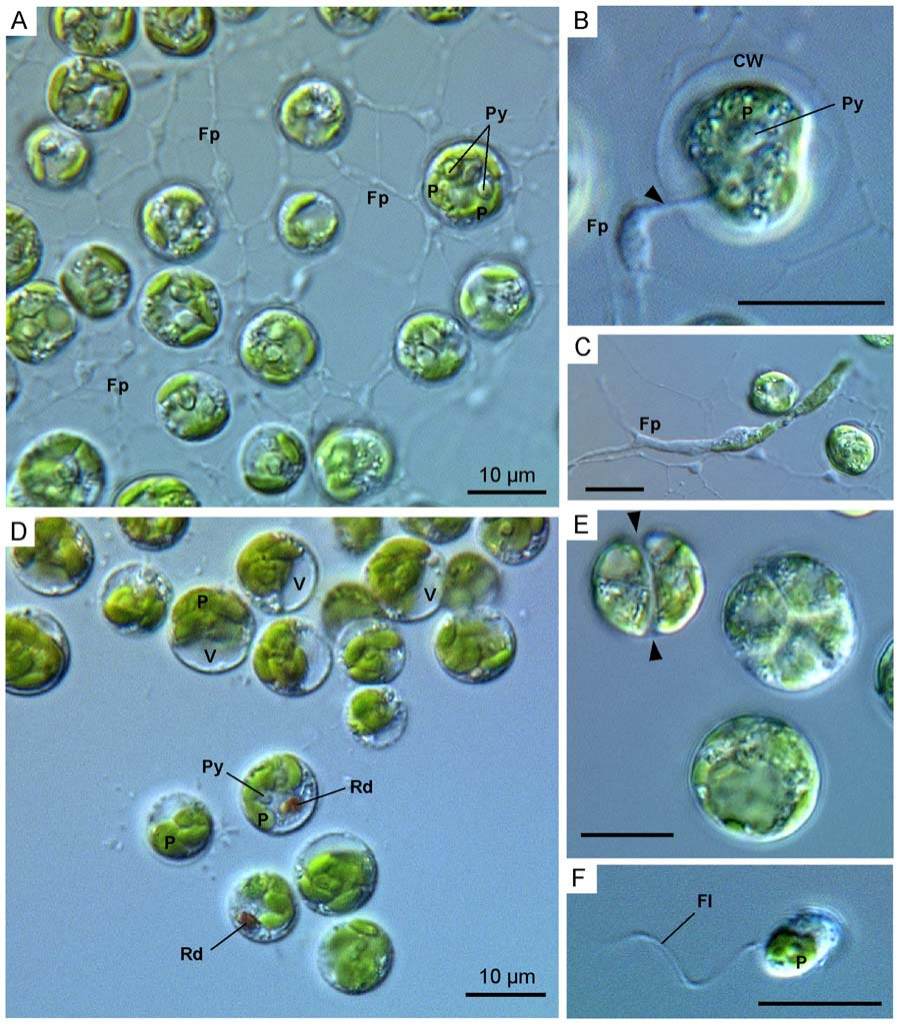
Differential interference contrast (DIC) micrographs of LEX01 strain. (A). Walled amoeboid cells in a fresh culture (3 days after the cells were transferred into new medium). (B). A walled amoeboid cell extending a filopodium through a pore of the cell wall (arrowhead). (C). Migrating amoeboid cell. (D). Walled coccoid cells in two weeks old culture. (E). Binary and quaternary cell divisions. Arrowheads indicate the parental cell wall. (F). Flagellate cell with a single flagellum and plastid. Scale bars are 10 µm. CW, cell wall; Fp, Filopodium; Fl, flagellum; P, plastid; Py, pyrenoid; Rd, reddish particle; V, vacuole.

**Figure 2:**
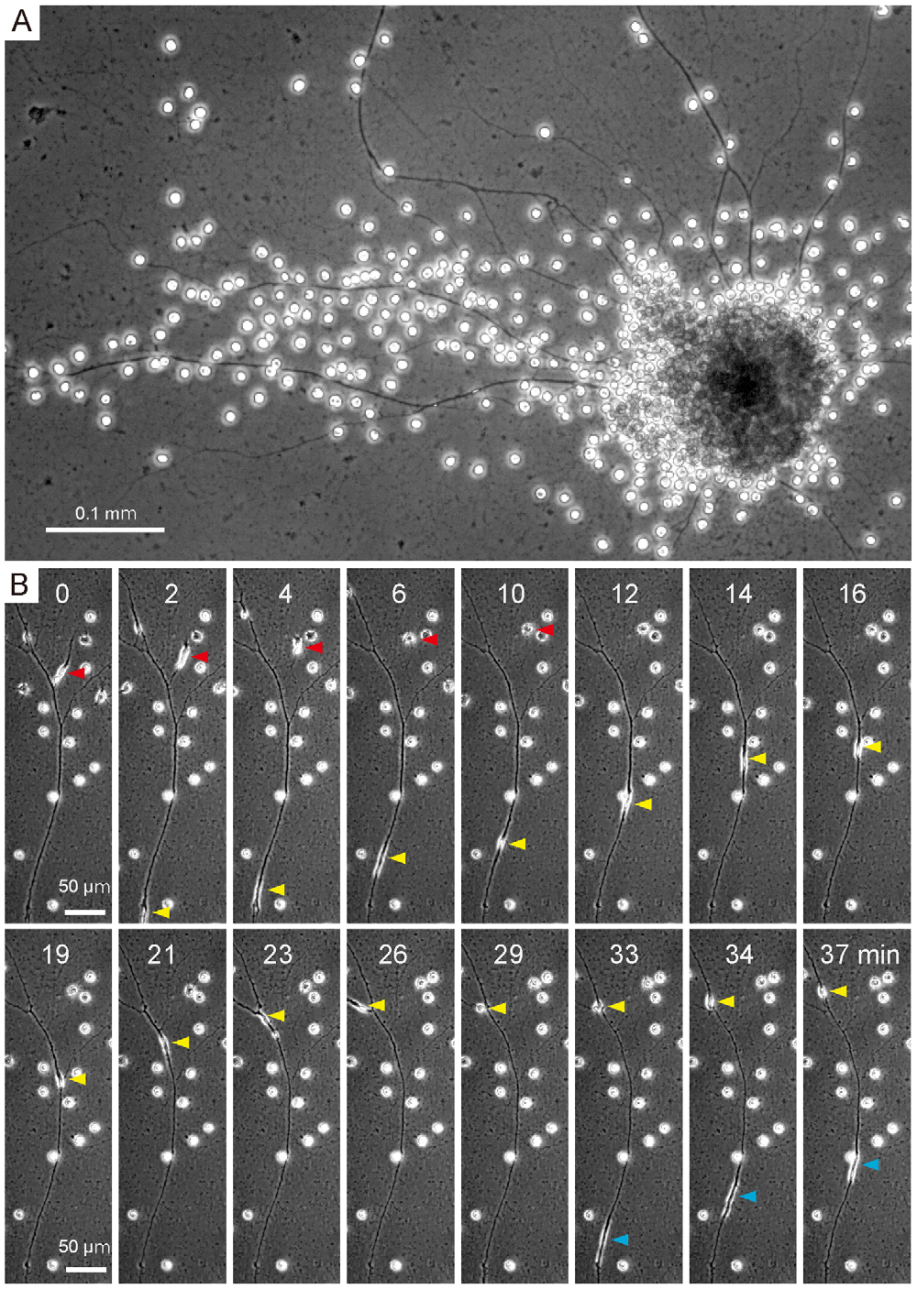
Colonization and migration behaviors of LEX01. (A) Long and thick filopodium extended from a large colony consisting of several hundred cells. (B) Time-lapse images showing the behavior of cells in a long and thick filopodia. Multiple naked amoeboid cells (arrowheads) moved in the filopodia from the colony (under) to the tip (upper). Each number of images indicates the time scale. These phase contrast images were taken by Axiovert 200 inverted microscope.

Molecular data show LEX01 to be a strain of *L. globosa* that is very closely related to the type strain, but its morphology was found to be significantly different. Most importantly, the life cycle of LEX01 strain was found to consist of four cell types by light microscopy: naked amoeboid, walled amoeboid, walled coccoid, and flagellate cells (Figure 1). In a fresh culture, 1 to 7 days after cells were transferred into new medium, walled amoeboid cells dominated (Figure 1A). These cells were 7.5 to 10.2 µm (mean = 8.4 µm, n = 30) in diameter (excluding filopodia). Each cell possessed two to five bilobate plastids having a bulbous pyrenoid, and extended filopodia through a pore in the cell wall (Figure 1B). Filopodia were interconnected, and formed wide reticulopodial networks (Figure 1A). A small number of naked amoeboid cells were also observed in the fresh culture, and they often formed a bipolar spindle shape (Figure 1C). Furthermore, we observed extremely long and thick filopodia, referred to as cytoplasmic strands [13], that were over 1 mm in length, and extended from a large colony consisting of several hundred cells (Figure 2A). Multiple naked amoeboid cells migrated in the cytoplasmic strands from the colony to the distal end with approximately 10–20 µm per minute, and then turned into walled amoeboid cells (Figure 2B, and Movie S1). This migration caused the cells to be dispersed in a roughly concentric pattern.

In contrast, walled coccoid cells, whose sizes were 6.0 to 11.8 µm (mean = 9.2 µm, n = 30) in diameter, dominated cultures after a couple of weeks (Figure 1D). The coccoid cells were tightly adhered to the bottom of a culture vessel, despite lacking filopodia, they often formed large colonies of several hundred cells. A vacuole and a reddish particle were sometimes observed in the cytoplasm (Figure 1D). The cells divided by binary or quaternary fission within the parental walls (Figure 1E). A couple of days after a walled coccoid culture was replenished by fresh medium, many flagellate cells appeared, with each flagellate possessing a single flagellum and a single plastid (Figure 1F).

### Ultrastructure of LEX01

**Figure 3:**
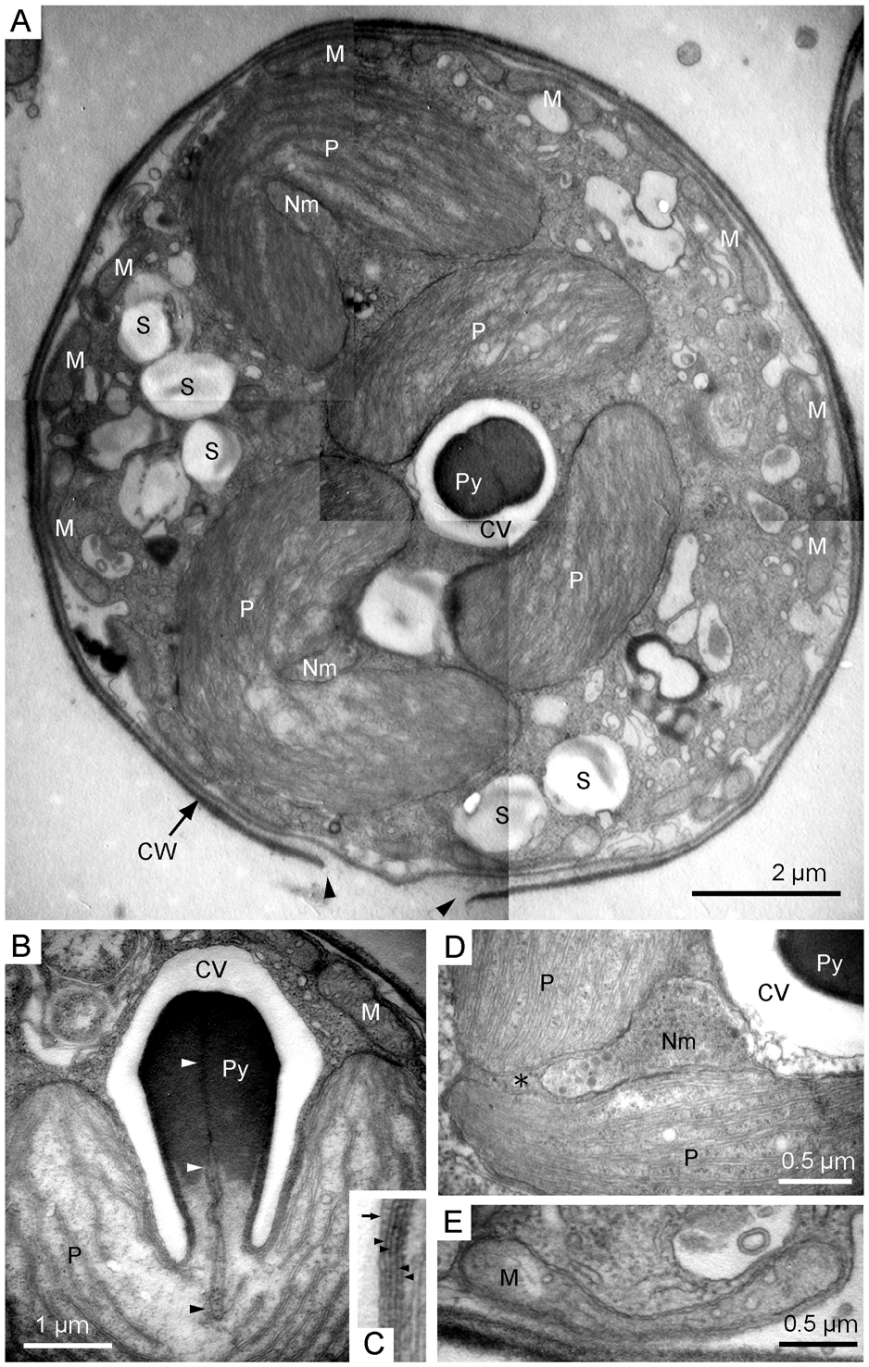
Transmission electron micrographs of LEX01 strain. (A). Walled coccoid cell showing general ultrastructure. This cell contains three bilobate plastids and several mitochondria that are seen near the single-layered cell wall. Arrowheads indicate the pore in the cell wall. (B). Longitudinal section of pyrenoid projecting from a plastid, showing an invagination of the inner pair of envelope membranes into the pyrenoid matrix (arrowheads). (C). Enlarged image of plastid membranes. An arrow and arrowheads indicate a capping vesicle membrane and four plastid membranes, respectively. (D). Section of periplastidal compartment (asterisk) containing a nucleomorph. (E). Tubular mitochondarion. CV, capping vesicle; CW, cell wall; M, mitochondrion; Nm, nucleomorph; P, plastid; Py, pyrenoid; S, storage metabolite vesicle.

To characterize the ultrastructure of LEX01, we observed the cells from a one week old culture by transmission electron microscopy. Each cell was bounded by a wall that was occasionally visible as a multilayer. Some sections revealed a pore in the cell wall (e.g., Figure 3A). The cells contained several plastids (Figure 3A), each of which was surrounded by four envelope membranes, and possessed a bulbous pyrenoid and a nucleomorph (Figure 3B, C, D). The pyrenoid was covered by a capping vesicle and invaginated longitudinally by the inner two membranes of the plastid, which reached the plastid stroma (Figure 3B). A single nucleomorph was located in the periplastidal compartment of each plastid, which corresponds to the space between the inner and outer pairs of plastid membranes, and was always located near the base of the pyrenoid (Figure 3D). Multiple mitochondria with tubular cristae were observed at the periphery of the cells (Figure 3A, E). Several vesicles containing storage product-like materials were also present in the cytoplasm (Figure 3A).

## Discussion

### Conflicting molecular and morphological identifications of LEX01

DNA barcoding indicated that LEX01 is a strain of *Lotharella globosa*, closely related to the type strain: although the conservation of nucleomorph ITS sequences is only 75.2 to 83.4% between *Lotharella* species, the nucleomorph ITS sequence of the LEX01 was found to be 100% identical to that of all three sampled strains of *L. globosa*. Other markers sampled tell the same story, meaning LEX01 cannot be distinguished from *L. globosa* based on such molecular characters. In contrast, however, the LEX01 strain is clearly discriminated from *L. globosa* based on morphological and ultrastructural characters that are traditionally used for chlorarachniophyte taxonomy. The most remarkable difference is that LEX01 possesses naked or walled amoeboid cells in its life cycle, while the type strain of *L. globosa* totally lacks such cells, which is indeed part of the formal description of this species [9]; we also reconfirmed that the type strain CCCM811 formed no amoeboid cell in the same condition as LEX01 culture (Figure S1). More subtle differences also exist: whereas LEX01 possesses an aperture in the cell wall through which extends a filopodium, the *L. globosa* cell wall lacks such an aperture, and vacuoles occasionally form in the cytoplasm of LEX01, but not *L. globosa*. We conclude that the LEX01 strain is a variety of *L. globosa*, and that different varieties of this species can have significant morphological differences. Even major morphological characteristics, such as the presence of life cycle stages, can be seen. This variation probably represents a lack of stability of these characters in natural populations.

#### Implications for other *Lotharella* species descriptions

In the genus *Lotharella*, amoeboid cells have been reported in *L. reticulosa*, *L. vacuolata*, and *L. polymorpha* (see Table 1 for a summary of features of all *Lotharella* species). These *Lotharella* species possess both naked and walled amoeboid cells whose filopodia are interconnected to form reticulopodial networks [5], [11], [13], as was also observed in LEX01. *L. reticulosa* resembles LEX01 in morphological characters in comparison with the other *Lotharella* species. Specifically, both LEX01 and *L. reticulosa* form clusters of coccoid cells (colonies) that radially extend many cytoplasmic strands (long and thick filopodia). Multiple cells migrate in the cytoplasmic strand from the colony to the distal end to be dispersed concentrically. Such colonization and migration behaviors have never been observed in other chlorarachniophyte species, and thus LEX01 and *L. reticulosa* are discriminated from *L. globosa* and *L. polymorpha* (Table 1). Although DNA barcoding clearly discriminates LEX01 from *L. reticulosa* with 83.4% identity of nucleomorph ITS sequences, morphologically there are few differences between them in a general culture condition. However, there are differences: *L. reticulosa* possesses some large cells (25–33 µm in diameter) and filopodial nodes (converged cytoplasmic strands between colonies) in old cultures [13], whereas such cells and structures have never been observed in LEX01 cultures. Our data therefore indicate that LEX01 is morphologically similar to *L. reticulosa*, but the DNA barcoding strongly indicates that it is *L. globosa*. There is a conflict between molecular and morphological comparisons among three *Lotahrella* strains, LEX01, *L. reticulosa*, and *L. globosa*.

**Table 1:** Comparison of morphological and ultrastructural characters among *Lotharella* species.

	LEX01 (this study)	*L. globosa* [9], [10]	*L. reticulosa* [13]	*L. oceanica* [12]	*L. vacuolata* [5]	*L. polymorpha* [11]
DNA similarity^1^		100%	83.4%	82.7%	75.2%	N/A
Main stage	Coccoid	Coccoid	Coccoid	Naked spherical	Coccoid	Coccoid
Flagellate cell	Present	Present	Present (rare)	Present	Present	Present
Amoeboid cell	Present	Absent	Present	Absent	Present	Present
Unique colony^2^	Present	Absent	Present	Absent	Absent	Absent
Intercellular migration	Present	Absent	Present	Absent	Absent	Absent
Cell wall^3^	Present (multiple)	Present (multiple)	Present (multiple)	Absent	Present (multiple)	Present (single)
Cell wall pore^4^	Present (large)	Absent	Present	Absent	Present (large)	Present (narrow)
Pyrenoid stalk	Present	Present	Present	Present	Present	Absent
Vacuole	Present	Absent	Present	Present	Present	Present
Quaternary fission	Present	Present	Absent	Absent	Absent	Absent

1 the number of “DNA similarity” shows sequence identity of nucleomorph ITS region between LEX01 and each species.

2 “Unique colony” means that cluster of cells extends cytoplasmic strands radially.

3 parentheses indicate the layer of cell wall.

4 parentheses of indicate the size of cell wall pore.

#### Loss of life cycle stages in chlorarachniophytes

**Figure 4:**
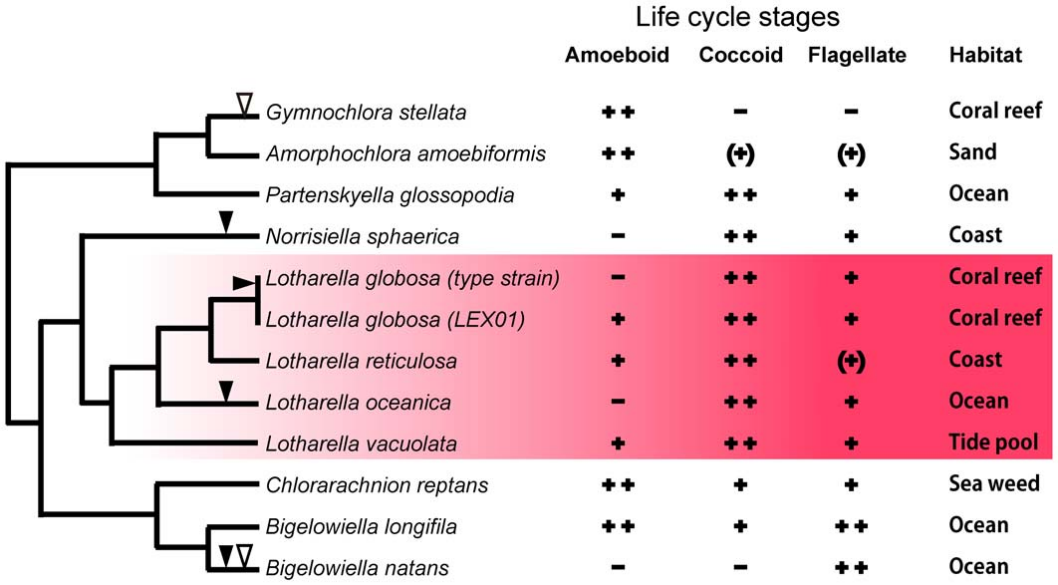
Characteristics of life cycles and phylogeny in chlorarachniophyte species. The tree was drew based on a phylogenetic tree of nucleomorph ITS sequences [13]. All information was obtained from [5]–[7], [9], [10], [12], [13], [16]–[19]. Each column shows present or absent of amoeboid, coccoid, and flagellate cells in life cycles. ++ indicates main stage; +, present; −, absent; (+), very rare. Solid arrowheads indicate losses of amoeboid stage in life cycles. Open arrowheads indicate losses of coccoid stage.

Half of the chlorarachniophyte species have been described as possessing all three major cell types (amoeboid, coccoid, and flagellate cells) in their life cycles, and half of them lack one or two cell types (Figure 4). For instance, the coccoid stage is absent in two distantly related species, *Bigelowiella natans* and *Gymnochlora stellata* [7], [10] (Figure 4). The most likely explanation is that a common ancestor of chlorarachniophytes possessed all three cell types, and species that lack any given stage have individually lost the ability to form that stage in the evolutionary history of this lineage. This suggests that life cycle stages have been lost on a number of occasions in chlorarachniophyte evolution, but until now this process had not been observed in the short term. Data we present here suggest that *L. globosa* strain LEX01 most likely represents the ancestral state of this species, whereas *L. globosa* CCMP1729 has lost the ability to form the amoeboid stage; their shared sequence identity of even the highly divergent ITS barcode suggests that this took place very recently (Figure 4).

Given that life cycle stages have been eliminated multiple times in divergent lineages of chlorarachniophyte species, one question that emerges is why do several chlorarachniophyte species lack the ability to form amoeboid cells? Ishida et al. (1999) [1] suggested that life cycles of chlorarachniophytes are associated with their habitats. Two planktonic species, *L. oceanica* and *B. natans*, were collected from open ocean, and neither possess an amoeboid form, which makes sense because these species are not required to or have no opportunity to attach to substrates (e.g. sand, seaweed, coral reef, etc.). This does not explain why a sessile species such as the type strain of *L. globosa* (CCMP1729), which was isolated from a coral reef, would lose the amoeboid stage – here the answer may be related to environmental stress. The formation of cell walls is believed to help resist environmental stresses such as rapid changes of temperature and salinity, so for example the amoeboid stage is very short in *L. vacuolata*, which has been isolated from a stressful environment [5]. Since coastal areas are susceptible to freshwater runoff, the absence of an amoeboid stage in the type strain of *L. globosa* (CCMP1729) might be adaptation to its habitat. Why another strain of *L. globosa* (LEX01) would still possesses an amoeboid stage, despite also being isolated from coral, is not clear.

#### Description: *Lotharella globosa* (Ishida et Y. Hara) Ishida et Y. Hara var. *fortis* Hirakawa et Keeling var. nov. (Figure 1, 2, and 3)

Diagnosis: *A typo differt cellulae nudae amoeboideae vel amoeboideae cum parietibus vel globosae cum parietibus vel flagellatae, parietibus cum foramen, cellulae vetus globosae cum vitalicirculo. Coloniae (fascicule cellularum) extensae filopodia radial.*

It differs from a type, cells naked amoeboid, walled amoeboid, walled coccoid, or flagellate, cell wall with a pore, old coccoid cell with vacuole. Colony (cell cluster) extending filopodia radially.

Holotype: One TEM block (accession number A88958), deposited in the Beaty Biodiversity Museum at the University of British Columbia (Canada).

Culture: LEX01 strain is maintained at the Canadian Center for the Culture of Microorganisms (accession number NEPCC920).

Type locality: Artificial coral reef at the Birch Aquarium, San Diego, CA, USA. 10 April 2010.

Etymology: The variety name *fortis* (vigorous) refers to the active migration of amoeboid cells.

Gene sequences: Nuclear SSU rRNA (accession number JF826444), nuclear ITS (JF806441 to JF806443), and nucleomorph ITS (JF806440) are deposited in GenBank.

## Materials and Methods

### Ethics Statement

Artificial coral reefs were provided from Fernando Nosratpour (assistant curator at the Birch Aquarium), and there are no permits needed for using them.

### LEX01 strain and culture conditions

LEX01 strain was isolated by Alexis Howe in April 2010 from an artificial coral reef at the Birch Aquarium (Scripps Institution of Oceanography, San Diego) that was provided from Fernando Nosratpour (assistant curator at the Birch Aquarium). It is deposited in the Canadian Center for the Culture of Microorganisms (accession number NEPCC920). The culture was maintained at 20°C under white illumination (55 to 60 µmol photons·m^−2^·s^−1^) on a 12∶12 hours light∶dark cycle in 6-well tissue culture plates or 250 mL polystyrene flasks containing ESM medium [15]. To observe cells under a light microscope, the cells were grown on a sterile coverslip that was placed into each well of the plate. To extract DNA, cells were cultured in the flask with 150 mL ESM medium. The strain CCCM811 was also cultured under the same condition with LEX01 for microscopic observation and DNA extraction.

### DNA extraction, PCR, and sequencing

The cells of *L. globosa*, LEX01 and CCCM811, were collected by centrifugation from 150 mL cultures and total DNA was extracted using DNeasy Plant Mini Kit (Qiagen) according to the manufacturer's instructions. Nuclear and nucleomorph internal transcribed spacer (ITS) regions between the LSU and SSU rRNAs were amplified from total DNA by PCR with Econotaq DNA polymerase (Lucigen) and specific primer sets designed by Gile et al. (2010) [8]. Primer sequences for the nuclear ITS regions were 5′-AACGAGGAATTTCTAGTAAAC-3′ (forward) and 5′-CAATCCCAAACAACACGACTCTTCG-3′ (reverse), and the nucleomorph ITS sequences were amplified using 5′-AACGAGGAATGCCTAGTAAGC-3′ (forward) and 5′-TCCTCCGCTTATTGATATGC-3′ (reverse). In addition, the nuclear small subunit (SSU) rRNA gene was amplified using universal primers 5′-GCGCTACCTGGTTGATCCTGCC-3′ and 5′-TGATCCTTCTGCAGGTTCACCTAC-3′. Each PCR product was purified using the QIAquick PCR Purification Kit (Qiagen). The nucleomorph ITS products were sequenced directly using Applied Biosystems 3730 DNA Analyzer with BigDye Terminator v3.1 cycle sequencing kit (Applied Biosystems). The nuclear SSU and ITS products were cloned into the pSC-A-amp/kan of the StrataClone PCR Cloning Kit (Stratagene), and three to five colonies were picked for sequencing. Plasmids were purified from *E. coli* using FastPlasmid Mini Kit (5 Prime), and then sequenced with M13 primers. All sequence data are deposited in GenBank (JF806440 to JF806448, JF826444, and JF826445).

### Light microscopy

Cells grown on a coverslip in 6-well tissue culture plates were fixed by 2.5% glutaraldehyde in ESM medium, and observed under an Axioplan 2 microscope (Carl Zeiss AG) with an 3CCD HD video camera XL H1S (Canon). To observe the behavior of naked amoeboid cells with extremely long filopodia, time-lapse images were taken under an Axiovert 200 inverted microscope (Carl Zeiss AG) with a MicroImager II digital camera (Qimaging); pictures were manually taken every 1 minute for an hour.

### Transmission electron microscopy

LEX01 cells were collected from 1 week old cultures, and the pellet was fixed in 2.5% glutaraldehyde in 0.2 M sodium cacodylate buffer (pH 7.2) with 2% NaCl for 2 hours at 4°C. The cells were washed three times with the sodium cacodylate buffer, and post-fixed in 1% osmium tetroxide for 2 hours at 4°C. The fixed cells were rinsed three times with the buffer, and dehydrated using an ethanol series (30 to 100%) followed by 100% acetone. After dehydration, the cells were infiltrated with 1∶1 acetone and JEMBED 812 resin mixture (Canemco & Marivac) for 6 hours, followed by two changes of 100% resin of 12 hours each at room temperature. The resin was polymerized for 16 hours at 70°C. The polymerized block was sectioned with an ultramicrotome (Leica EM UC6), and ultra thin sections were collected onto Formvar-coated copper grids. The grids were stained with uranyl acetate and lead citrate for 5 to 10 minutes, and observed under a Hitachi H7600 electron microscope at 80 kV.

## Supporting Information

Movie S1 **Time-lapse movie of naked amoeboid behavior of LEX01.**
(MWV)

Figure S1 **Comparative observation between two strains of** ***Lotharella globosa*** **.** On the left and right are three DIC micrographs of LEX01 and CCCM811 strains, respectively. These images were taken 3 and 14 days after the cells were transferred into new medium. Both LEX01 and CCCM811 strains were cultured under the same condition: the same medium, temperature, light intensity, cell density, and type of culture dish.
(TIF)
