# Supplementary figures and images for: Morphological Diversity between Culture Strains of a Chlorarachniophyte, Lotharella globosa
Source: PLoS One. 2011 Aug 15;6(8):e23193. doi: 10.1371/journal.pone.0023193 (PMC3156133; doi:10.1371/journal.pone.0023193)

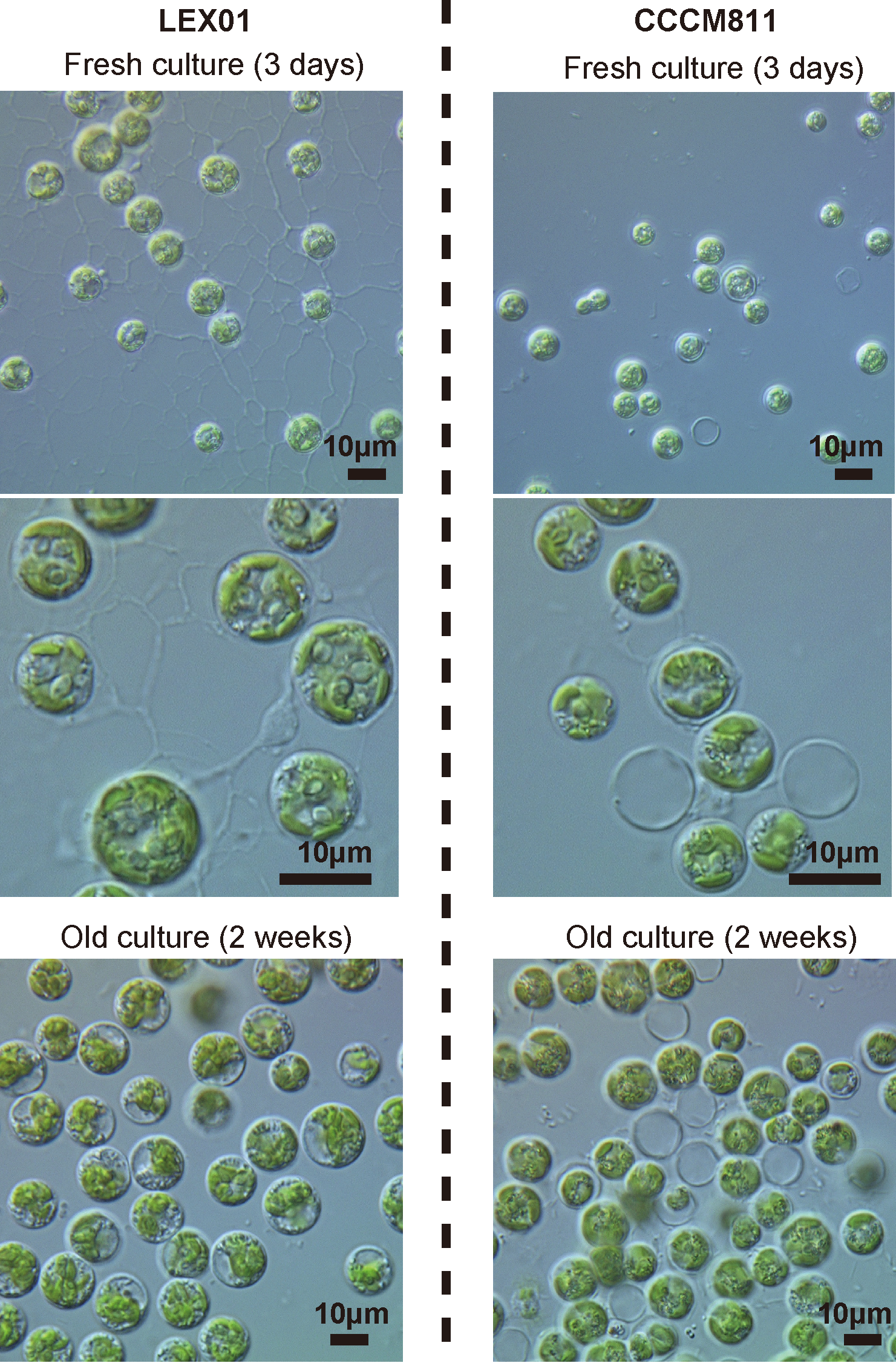

Supplement: Figure S1 — Comparative observation between two strains of Lotharella globosa . On the left and right are three DIC micrographs of LEX01 and CCCM811 strains, respectively. These images were taken 3 and 14 days after the cells were transferred into new medium. Both LEX01 and CCCM811 strains were cultured under the same condition: the same medium, temperature, light intensity, cell density, and type of culture dish. (TIF) [file pone.0023193.s002.tif]
